# Comparison of heat production and bone architecture changes in the implant site preparation with compressive osteotomes, osseodensification technique, piezoelectric devices, and standard drills: an ex vivo study on porcine ribs

**DOI:** 10.1007/s10266-022-00730-8

**Published:** 2022-07-19

**Authors:** Nishith Bhargava, Vittoria Perrotti, Vito Carlo Alberto Caponio, Victor Haruo Matsubara, Diana Patalwala, Alessandro Quaranta

**Affiliations:** 1 Dental School, University of Western Australia, Perth, WA Australia; 2 Deptartment of Medical, Oral and Biotechnological Sciences, G. D’Annunzio University of Chieti-Pescara, Via dei Vestini, 31, 66100 Chieti, Italy; 3 Department of Clinical and Experimental Medicine, University of Foggia, Foggia, Italy; 4 Dental School, University of Western Australia, Perth, WA Australia; 5 Centre for Microscopy, Characterization and Analysis, The University of Western Australia, Perth, WA Australia; 6 School of Dentistry, University of Sydney, Sydney, NSW Australia; 7 Scientific and Education Director, Smile Specialists Suite, Newcastle-Sydney, NSW Australia

**Keywords:** Dental implants, Drills, Micro-CT, Osseodensification technique, Osteotomes, Primary stability, Piezoelectric device

## Abstract

This study aimed at investigating differences in heat generation and bone architecture following four different implant site preparation techniques: compressive osteotomes, conventional drills, osseodensification (OD mode with osseodensification drills), and piezoelectric systems. Porcine rib bones were used as a model for implant surgery. Thermocouples were employed to measure temperature changes, and micro-CT to assess the bone architecture. The primary stability and insertion torque values of the implants placed in the differently prepared sites were assessed. The temperature changes were higher with Piezo. The average primary stability using the ISQ scale was the greatest for drills (76.17 ± 0.90) and the lowest for osteotomes (71.50 ± 11.09). Insertion torque was significantly higher with the osseodensification method (71.67 ± 7.99 Ncm) in comparison to drills, osteotomes, and piezo. Osteotomes showed the highest bone to implant contact percentage (39.83 ± 3.14%) and average trabecular number (2.02 ± 0.21 per mm), while drills exhibited the lowest (30.73 ± 1.65%; 1.37 ± 0.34 per mm). Total implant site bone volume was the highest with osseodensification (37.26 ± 4.13mm^3^) and the lowest for osteotomes (33.84 ± 3.84mm^3^). Statistical analysis showed a high primary stability and decrease in temperature during implant site preparation with osseodensification technique. The results support the use of osseodensification technique for implant site preparation.

## Introduction

Dental implants have emerged as an important and viable solution for oral rehabilitation with the advancement in technology and research. The process of osseointegration is complex and may be affected by several factors, which include thermal and mechanical changes that occur at the time of implant site preparations [1]. Indeed, it is reported that the heat generated during the implant site preparation can have a negative impact on the maturation of bone tissue at the bone/implant interface and in turn diminish the chances of osseointegration [2]. Therefore, controlling the amount of heat generated during the preparation phase is essential for osseointegration [3]. In previous studies, it was revealed that temperatures higher than 47 °C for about 1 min can result in osteonecrosis [4]. Indeed, heat impacts the bone tissue by causing hyperemia, necrosis, fibrosis, osteocytic degeneration, increased osteoclastic activity [3, 5], denaturation of alkaline phosphatase [6, 7], and dislocation of the structure of hydroxyapatite mineral lattice [5, 8]. During the drilling step, the friction between the drill and the bony wall raises the bone temperature; this heat is proportional to the pressure applied and the time of drilling [9]. Eriksson and Adell [10] suggested the use of low hand pressure while drilling to avoid bone overheating. However, the amount of pressure applied cannot be standardized due to the human factor [11].

There are many other variables such as the bur shape, sharpness and wear, speed of the drill, applied axial load, and the density of the bone that can affect the amount of heat generated at the time of implant site preparation [7, 12]. Stelzle et al. [13] compared the amount of heat generated by a spiral bur, a trephine bur or piezoelectric surgery. They applied an increasing load between 0 and 1000 g and concluded that temperature increased with the increase of the pressure when the trephine burs or piezoelectric surgery was used. However, they observed that the temperature decreased at a load of 500 g when a conventional drill was employed. Finally, they defined a maximum load of 100–400 g for piezosurgery, 100–200 g and 500–1000 g for spiral drill, and 100–600 gms for the trephine bur. The design and shape of the drill can also influence the implant bed preparation as they might have some effect on the heat generation. The drills are usually of the same shape as that of the implant. For the preparation of the implant bed twist drills are used for screw shaped implants and triflute drills for cylindric implants [14]. Oh et al. [15] conducted a pilot study to evaluate the amount of heat produced with different drills’ designs. They introduced modifications in the conventional triflute drill by reducing the diameter and setting the lateral cutting surface and used conventional triflute drills as control. They concluded that a lower amount of heat was produced when the area of the contact between drill and bone was reduced. More recently, it was shown that the rise in temperature is inversely proportional to the drill diameter. Strbac et al. observed that a 2 mm diameter twist drill reached a higher temperature than 3.5 mm diameter conical drill [16]. Regarding the role of the drilling speed in the generation of heat during the preparation of the bone bed for implant placement, it was concluded that low speed hand pieces (speed between 1500 and 2000 rpm) do not cause any impaired bone regeneration [17, 18]. The condition of the drill, particularly the wear and the sharpness, is another important factor in the regulation of temperature during drilling. The sharpness of the bur depends on the number of times it has been used, on the pressure applied during the implant site preparation, on the sterilization techniques, bone density and surface treatment [9]. Much higher temperatures were recorded when a worn drill was used [19]. Finally, it is also important to consider the type of bone present; indeed, the thermal conductivity of the bone is different for the cortical and the cancellous bone because of different rates of vascular penetration; in the cancellous bone it is about 0.5 mm/day whereas in the cortical bone it is about 0.05 mm/day. This means that the thermal conduction is better for the cancellous bone and the cortical bone is better for the initial implant stability [20].

Heat production and bone changes during implant site preparation can greatly vary depending on which technique is used. Three different techniques are commonly used in implant site preparation: osteotome, conventional drilling, and piezoelectric surgery. A few studies have focused on the heat generated when piezoelectric systems are used. According to Rashad et al. [21] piezoelectric systems produced more heat than the conventional drilling, exceeding the temperature of 47 °C even with irrigation. Although using piezoelectric devices is a gentle method of bone cutting, it has a higher mean temperature and is more time consuming when compared to conventional drilling, especially when the osteotomies are performed in dense bone [13, 22, 23]. Recently, a new technique has been introduced in the market to increase the density of trabecular bone around implants, called osseodensification technique [24]. Osseodensification is a non-excavation osteotomy preparation method introduced to improve the primary stability of implants placed in low-density bone sites [25]. In contrast to traditional standard drilling, this approach involves the use of specially designed densifying burs that have four or more lands and flutes that smoothly compact and auto-grafts bone into open marrow spaces [26], increasing the bone density—if utilized in counter-clock wise mode—and implant insertion torque through the preservation and densification of osteotomy site walls.

Though there is ample documentation of information on the temperature that is generated during preparation of implant sites, there are no studies available in the literature comparing all these different techniques; therefore, the present investigation was aimed at analyzing the differences in heat generation and changes in bone architecture during implant site preparation between compressive osteotomes, drills, osseodensification technique, and piezoelectric surgery to define which technique can be safely applied to achieve the necessary implant primary stability without damaging the surrounding bone.

## Materials and methods

The present investigation does not require ethical approval as it is an ex vivo study on porcine bone ribs. Guidelines for assessment of bone microstructure in rodents using micro-computed tomography have been followed for the images, procedure/method, results, Terminology/definitions [27].

Four different techniques—conventional compressive osteotomes, osseodensification technique using Osseodensification burs, piezoelectric surgery technique, and conventional drilling protocol—were used to prepare 60 implant sites (15 per group) on porcine ribs to assess the temperature changes. Implant sites’ preparation was performed by a single experienced operator (NB) using brand new instruments till 12 mm length and 4 mm width was reached.

**Fig. 1:**
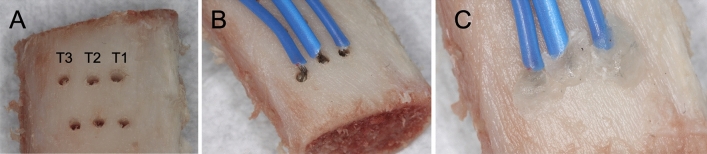
Thermocouples for temperature changes assessment. **A** Canals prepared at three different levels (T1, T2, T3) from the crest of porcine ribs. **B** Thermocouples were connected to a digital thermometer. **C** Insulation of thermocouples achieved with sticky wax at the canal opening

The porcine ribs were stored at − 4 °C for 1 week. The ribs were taken from pigs that were approximately 6 months old. The inclusion criteria for the ribs were as follows: (1) more than 15 mm in length and 6 mm in width; (2) presence of approximately 1.5–2 mm of cortical bone, which is similar to D2 bone according to the Lekholm and Zarb classification [28]. Eight hours before the start of implant site preparation, they were immersed in water at 36 °C (baseline temperature) for 2 h. Custom-made screw assisted metal holders secured the specimens during the osteotomy protocol. Three canals for thermocouples at three different depths (2 mm: T1–cortical bone, 7.5 mm: T2–cancellous bone, and 10.5 mm: T3–deep cancellous bone) from the crest of the rib were drilled using 1.5 mm twist drill perpendicularly to each implant site preparation and using intermittent cutting to a distance approximately 1 mm from the planned osteotomy site (Fig. 1A). The thermocouples were secured to the bone blocks at a distance of approximately 1 mm from the future osteotomy and insulated by the use of sticky wax at the canal opening (Fig. 1B, C). The thermocouples were connected to a digital thermometer that allowed a continuous temperature reading, and temperature changes (baseline and final temperature readings) during the osteotomy preparation were recorded for statistical analysis.

For the micro-CT experiments the ribs were prepared using a similar protocol as described above, but no thermocouples were inserted. Bone architecture analysis was conducted before and after the placement of a total of 24 (6 per group) parallel walled implants (4.3 mm × 12 mm—Southern Implants, Irene, South Africa) with a tri-lobe connection to assess the primary stability and changes in bone microstructure following implant placement.

### Osteotome group (Osteotomes)

Surgical compressive osteotomes (Southern Implants, Irene, South Africa) 2 mm, 3 mm, and 4 mm wide with concave tips were used for site preparation. The cortical plate, initially, was perforated using a surgical round bur. Subsequently, the sharpened tip of the osteotomes allowed for the preparation of the implant site through the condensation and cut of the trabecular bone up to the point where the planned depth was achieved. The site was subsequently enlarged through the activation of various osteotomes using a surgical mallet in a cumulative manner and increasing progressively the size until the planned diameter was reached (4 mm). The operation time was 6–7 min.

### Osseodensification group (Osseodensification)

**Fig. 2:**
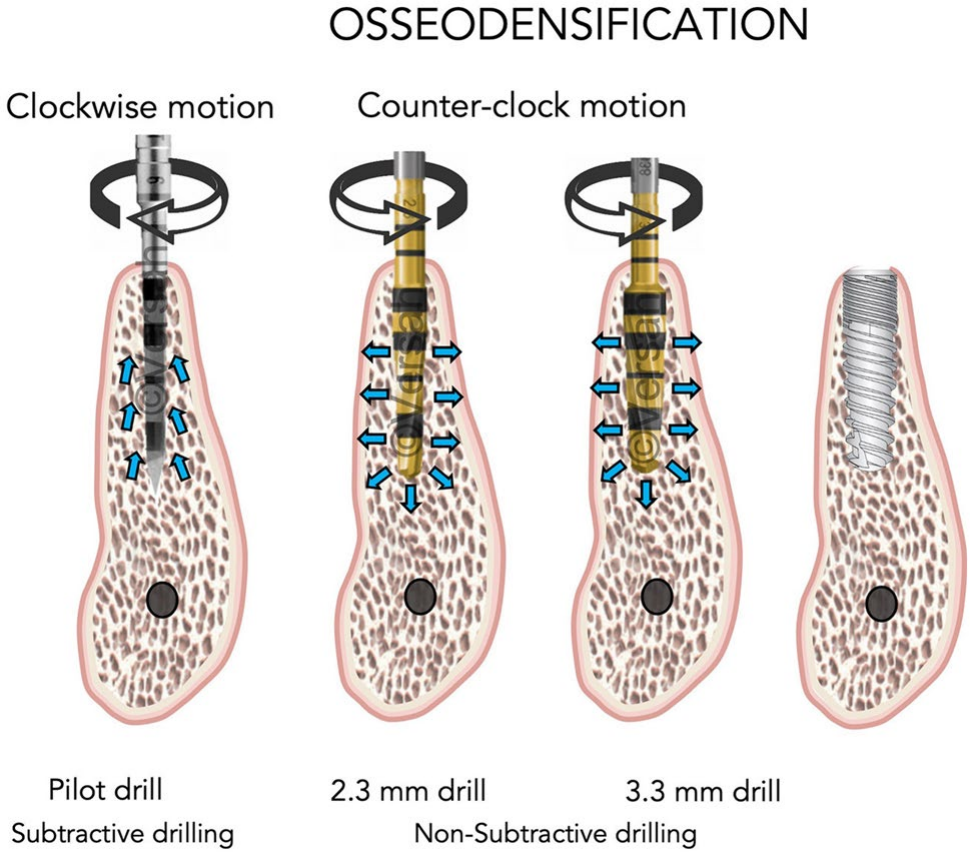
Drilling technique applied in the osseodensification group: pilot drill was applied in a clockwise direction to perforate the cortical bone. The subsequent drills were used in counter-clock direction at 1100 rpm until the desirable length of the osteotomy was achieved. (Courtesy Versah, LLC. ^©^ Used with Permission)

A pilot drill (Versah, Jackson, MI, USA) was used in a clockwise direction for the initial osteotomy, where the bony structure is removed. This was followed by 2.3 mm (VT1828) and 3.3 mm (VT2838) drills used in a counter-clock direction at 1100 rpm to achieve the desired length of the osteotomy, with copious irrigation as per the manufacturer’s recommendations (Fig. 2). The use of osseodensification bur in counter-clock motion creates non-subtractive drilling that is claimed to preserve and condense bone during the implant site preparation. The operation time was 5–6 min.

### Piezoelectric surgery group (Piezo)

An NSK piezoelectric surgery unit (NSK, Tokyo, Japan) was used with the corresponding implant site preparation kit under copious irrigation. A surgical round bur was used to perforate cortical bone plate. This was followed by drilling and widening the osteotomy in a sequential manner using a sequence of piezo drills tips (0.9 mm, 1.3 mm and 2 mm diameter) to reach the predetermined depth of 12 mm. The operation time was 7–8 min.

### Conventional drill group (Drills)

Implant sites were prepared using a series of drills (Southern Implants, Irene, South Africa) and gradually increased their size under copious irrigation, as per the manufacturer’s recommendations. To initiate osteotomy D-3Spade drill was used at 1100 rpm, followed by pilot a drill 2.0, then 3.0 and finally a 3.3 to the predetermined length of 12 mm. The operation time was 5–6 min.

### Primary stability assessment

A total of six implants were placed manually in each study group. Their primary stability was measured using Resonance Frequency Analysis (RFA-Neoss Penguin^RFA^ system) as per the manufacturer’s instructions. Implant-specific smart pegs (MulTipeg-13) were used for the readings and results were given as the measurement unit implant stability quotient (ISQ). Two ISQ readings were taken per implant (opposite sides, perpendicular to the smart peg). The insertion torque of implants placed in the group were also measured using a hand torque wrench.

### Bone architecture assessment

The porcine ribs were imaged twice before and after the implant placement (6 samples per group) with a high-resolution micro-CT scanner (Bruker Skyscan 1176, Kontich, Belgium). The ribs were placed on a polystyrene insert (inner diameter = 3.4 cm and outer diameter = 6.8 cm), and then placed on a carbon-fiber rat bed. They were scanned at 17.78 um pixel size with a 0.1 mm thick copper filter at 90 kV voltage, 278 µA current and a 560 ms scanning time for each specimen. The acquisition settings used were rotation step of 0.3° between each raw projection, a complete 360° scan and a frame averaging of 3. The acquired data were reconstructed by Bruker Skyscan NRecon software (v1.7.1.0) using a modified Feldkamp cone-beam algorithm. Reconstruction settings used were smoothing—2, Ring Artefact Correction—8 and Beam Hardening Correction—30%. The lower and upper threshold limits in attenuation coefficients were − 0.003 and 0.05, respectively, and were kept constant for all samples.

**Fig. 3:**
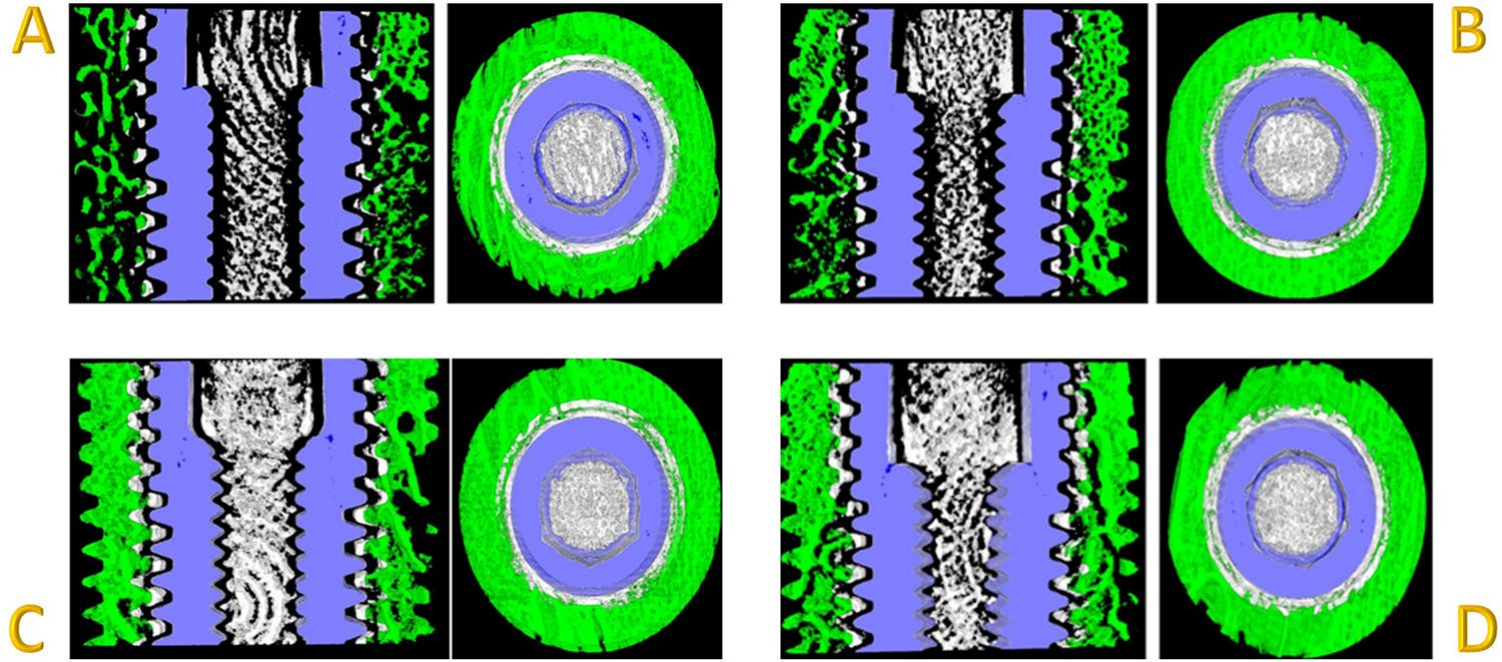
Bone architecture assessment. **A** Osteotomes, **B** Osseodensification, **C** Piezo, and **D** Drills. The green area represents bone analyzed, blue area represents implant and white area represents area left to compensate for beam hardening

Bruker Skyscan CT analyser software (v1.17.7.2) was used for the analysis with a custom-developed workflow based on the Bruker Micro-CT Method Note MN074—Bone around metal implant 3D-2D (Bone Implant Contact). The volume of interest (VOI) consisted of 300 slices, with the implant site/implant surrounded by trabecular bone and the reference slice being the one completely surrounded by bone (no air interface). An offset of 24 pixels (0.36 mm) was left from the implant surface to avoid any beam hardening artifacts caused by the metal implant. The circular volume of interest around the implant to study the peri-implant bone was 56 pixels wide (1.008 mm). For bone-implant contact analysis, we took into factor the 2D Intersection Surface (i.S; mm^2^) (or Bone Implant Contact surface area) and the Percent Intersection Surface (or Bone Implant Contact percentage). For the 3D analysis, the percentage of bone volume per tissue volume and the trabecular number (Tb.N; 1/mm) were quantified. The Tb.N implies the number of traversals across a trabecular or solid structure made per unit length on a random linear path through the VOI (Fig. 3A–D).

### Statistical analysis

To our knowledge, this is the first study addressing a multiple comparison among 4 different techniques. To calculate sample size, we referred to a previously published paper investigating 3 different techniques on porcine ribs [29].

Primary stability was considered the main outcome and sample size was calculated following 2 different methods: first, from the previously published study we calculated population variance and error which were input in STATA power one-way command to calculate sample size with a power of 90% and *p* value = 0.05. The command returned a total sample size of 12, 3 in each of the 4 groups.

Secondly, the sample size was calculated to find a statistically significant difference with a 90% power and *p* value = 0.05 between drills and compactors from the same previously published paper [29]. Each group mean and S.D. were input in STATA command power two means, which returned a total sample size of 24, 12 each group. In this study were included 15 samples each group.

Raw data are available on request. Mean ± standard deviation (SD) from three independent experiments are expressed. One-way ANOVA test followed by the nonparametric analysis Fisher’s least significant difference (LSD) was used to compare the bone temperature variations, primary stability, insertion torque, and trabeculae number among groups. All analyses were performed using SPSS Statistics and *P* *˂0.05* was considered statistically significant.

## Results

### Temperature changes

**Fig. 4:**
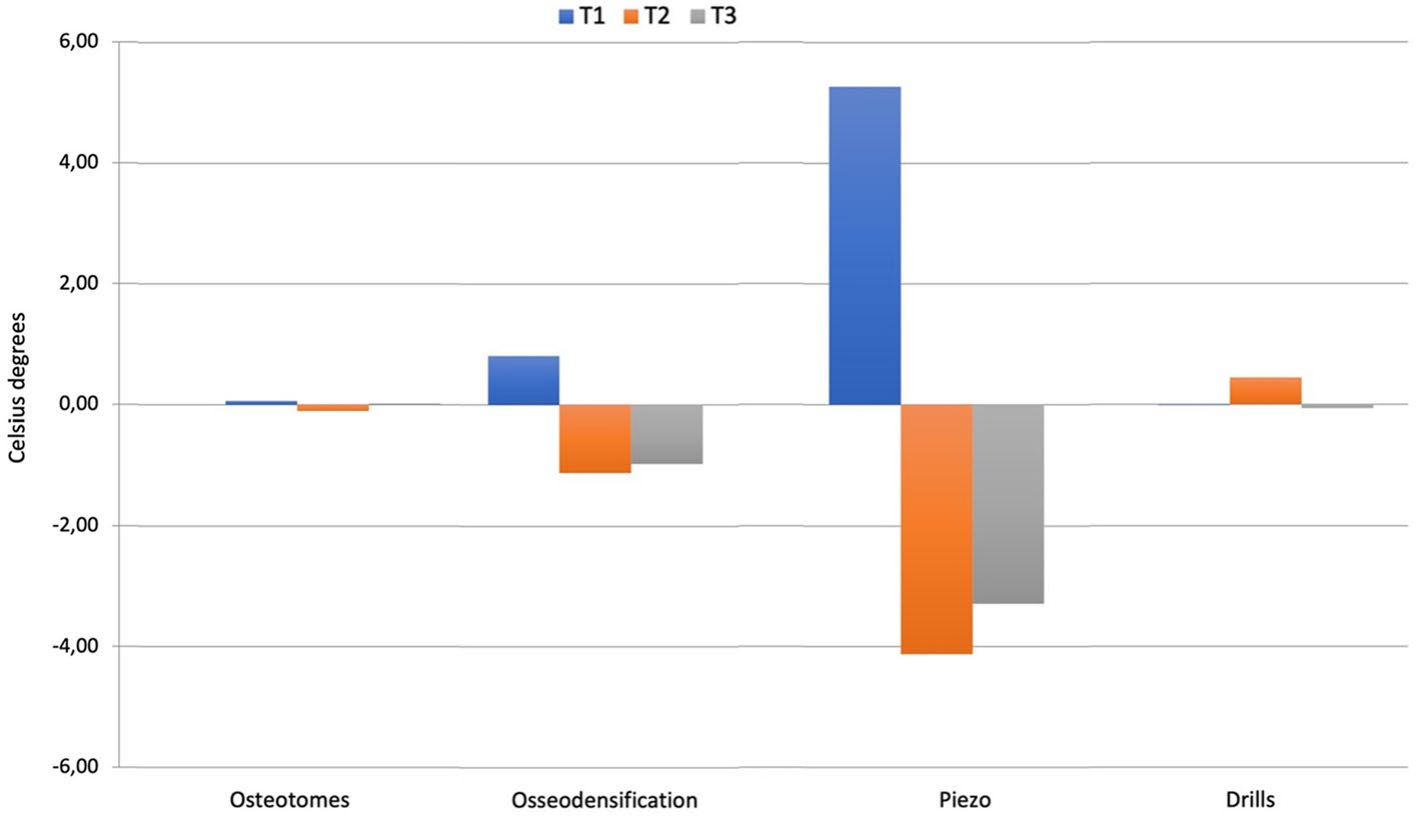
Mean temperature (°C) difference at T1 (2 mm depth), T2 (7.5 mm depth), and T3 (10.5 mm depth) in all the four groups

At T1 site, the piezoelectric surgery group showed the highest change of temperature (5.27 ± 0.88 °C) followed by the osseodensification (0.81 ± 0.71 °C), osteotomes (0.07 ± 0.48 °C), and conventional drills (− 0.01 ± 0.54 °C) groups with significant differences among all groups except for the comparison between conventional drills and osteotomes. The mean temperature changes at T2 site were 4.13 ± 1.53 °C for piezo followed by the conventional drill (0.45 ± 0.79 °C), osteotomes (− 0.11 ± 0.40 °C), and osseodensification (− 0.11 ± 0.40 °C). Only the comparisons between piezoelectric device and conventional drill and between osteotomes and osseodensification achieved the statistical significance. At T3 site the mean temperature changes were 3.29 ± 1.59 °C for piezoelectric device followed by osseodensification (− 0.97 ± 1.28 °C), conventional drills (− 0.06 ± 0.96 °C), and osteotomes (0.03 ± 0.96); as at T1 also at T3 only the temperature changes between osteotomes and drills were not statistically significant (Table 1A, B and Fig. 4).

**Table 1:** (A) Mean values and standard deviations of the temperatures registered for the four techniques at the different depths (T1, T2, T3); (B) Post hoc comparison among the four different techniques, regarding the temperatures generated at T1, T2, T3 depths

A Groups	Mean ± standard deviation		
	T1	T2	T3
Osteotomes	22.92 ± 1.68	22.73 ± 1.89	22.82 ± 1.76
Osseodensification	24.33 ± 0.99	21.31 ± 0.81	21.41 ± 0.80
Piezo	29.53 ± 1.00	15.98 ± 1.84	16.62 ± 2.01
Drills	22.89 ± 1.10	23.31 ± 0.79	23.42 ± 1.36

B Compared group	Reference group	Mean difference (T1)	Mean difference (T2)	Mean difference (T3)
Osteotomes	Osseodensification	− 0.82*	0.55	0.91^*^
	Piezo	− 5.28*	4.57^*^	3.23^*^
	Drills	0.08	− 0.55	0.09
Osseodensification	Osteotomes	0.74*	0.00	− 0.99*
	Piezo	− 4.46*	4.02^*^	2.32^*^
	Drills	0.82*	− 0.55	− 0.91*
Piezo	Osteotomes	5.20*	− 4.02*	− 3.32*
	Osseodensification	4.46*	− 4.02*	− 2.33*
	Drills	5.28*	− 4.57*	− 3.23*
Drills	Osteotomes	− 0.08	0.55	− 0.09
	Osseodensification	− 0.82*	0.55	0.91^*^
	Piezo	− 5.28*	4.57^*^	3.23^*^

^*^Indicates the statistically significant values (*p* < 0.05). *Indicates statistically significant values (*p* < 0.05). Positive mean difference = Decrease in insertion torque from the compared group to the reference group. Negative mean difference = Increase in insertion torque from the compared group to the reference group

### Stability

In terms of RFA, no statistical difference was found among all four modalities (Table 2).

**Table 2:** Mean values of ISQ scores after resonance frequency analysis for the four groups

Groups	Mean ± standard deviation
Osteotomes	71.5 ± 11.09
Osseodensification	73.92 ± 11.22
Piezo	71.67 ± 12.53
Drills	76.17 ± 0.90

*P* value = 0.836

Regarding the insertion torque, implants inserted following the osseodensification protocol exhibited the highest insertion torque (71.67 ± 7.99 Ncm) followed by the piezo device (43.33 ± 3.73 Ncm), conventional drills (35 ± 0.00), and finally osteotomes (31.67 ± 5.53). When all the groups were compared using the one-way ANOVA followed by post hoc least significant difference (LSD) test, it was seen that the osseodensification group and the piezo group had significantly higher insertion torque than the others, and the drills and the osteotomes were not statistically different from each other (Table 3 A, B).

**Table 3:** (A) Mean values and standard deviations of the insertion torques registered for the four techniques; (B) Post hoc comparison among the four different techniques, regarding the insertion torques

A Groups	Mean ± standard deviation
Osteotomes	31.67 ± 5.53
Osseodensification	71.67 ± 7.99
Piezo	43.33 ± 3.73
Drills	35.00 ± 0.00

B Compared group	Reference group	Mean difference (Ncm)
Osteotomes	Osseodensification	− 40.00*
	Piezo	− 11.67*
	Drills	− 3.33
Osseodensification	Osteotomes	40.00*
	Piezo	28.33*
	Drills	36.67*
Piezo	Osteotomes	11.67*
	Osseodensification	− 28.33*
	Drills	8.33
Drills	Osteotomes	3.33
	Osseodensification	− 36.67*
	Piezo	− 8.33

^*^Indicates statistically significant values (*p* < 0.05). Positive mean difference = Decrease in insertion torque from the compared group to the reference group. Negative mean difference = Increase in in insertion torque from the compared group to the reference group

### Bone architecture assessment

#### Trabeculae number

The mean trabeculae number was the highest for osteotome (2.02 ± 0.27/mm) followed by piezo (1.76 ± 0.37/mm), osseodensification (1.72 ± 0.27/mm), and Drill (1.37 ± 0.37/mm). Indeed, the group in which the osteotomy was performed using conventional drills showed a statistically significant lower trabeculae number when compared to the site prepared using an osteotome, while no significant differences were found among the other groups (Table 4A, B).

**Table 4:** (A) Mean values and standard deviations of the trabeculae numbers for the four techniques; (B) Post hoc comparison among the four different techniques, regarding the trabeculae numbers

A	Mean ± standard deviation
Osteotomes	2.02 ± 0.27
Osseodensification	1.72 ± 0.27
Piezo	1.76 ± 0.37
Drills	1.37 ± 0.37

B Compared group	Reference group	Mean difference
Osteotomes	Osseodensification	0.31
	Piezo	0.26
	Drill	0.66*
Osseodensification	Osteotome	− 0.31
	Piezo	− 0.043
	Drill	0.35
Piezo	Osteotome	− 0.26
	Osseodensification	0.04
	Drill	0.40
Drills	Osteotome	− 0.66*
	Osseodensification	− 0.35
	Piezo	− 0.40

^*^Indicates statistically significant values (*p* < 0.05). Positive mean difference = Decrease in trabeculae number from the compared group to the reference group. Negative mean difference = Increase in trabeculae number from the compared group to the reference group

#### 3D bone volume

**Fig. 5:**
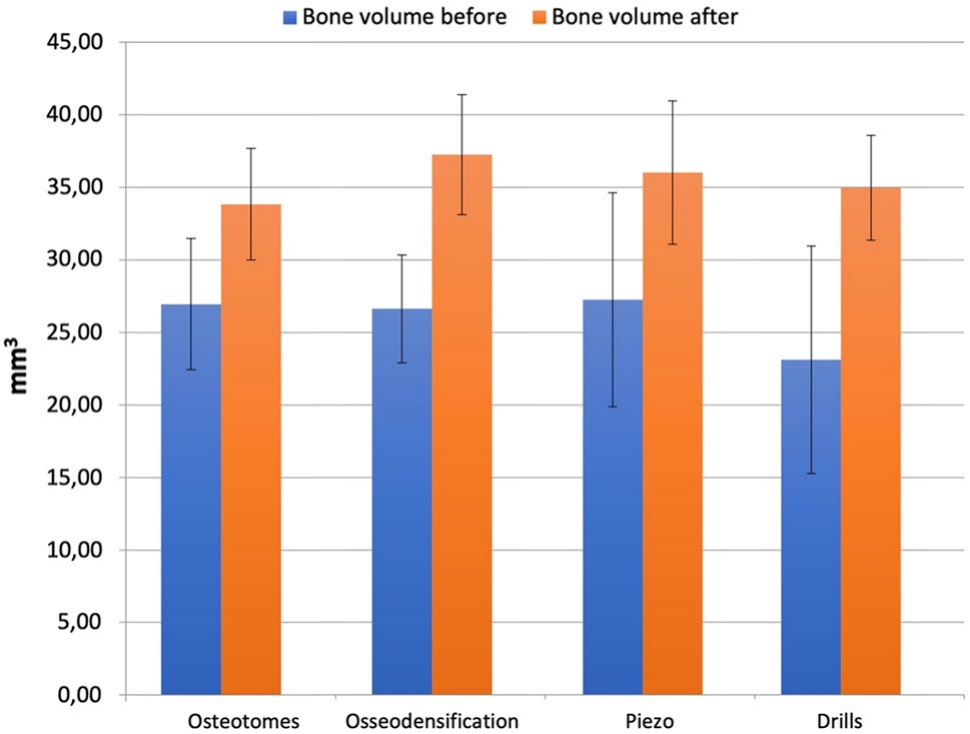
Bone volume before and after implant placement

**Fig. 6:**
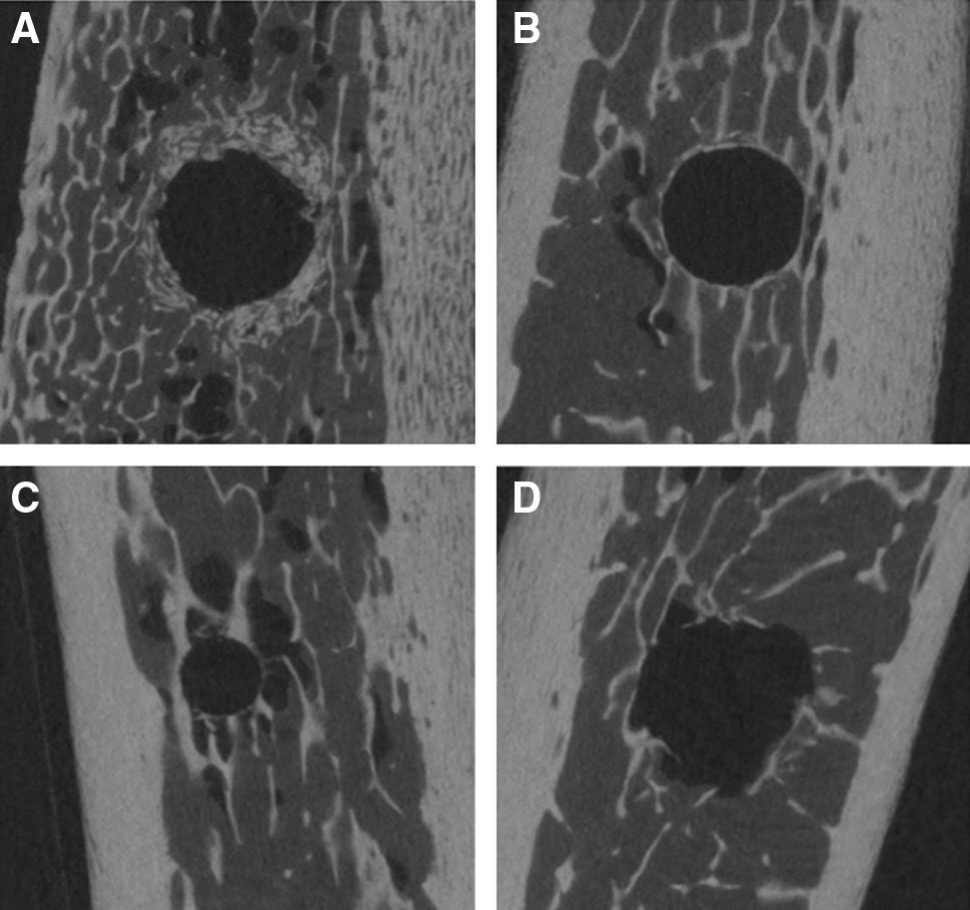
Micro-CT image showing bone patterns around osteotomies prepared using different modalities. **A** Osteotomes, **B** osseodensification, **C** piezosurgery, and **D** conventional drills

All four methods produced a significant increase in bone volume after the implant placement, although no statistical difference was observed among groups (Fig. 5). According to Micro-CT images (Fig. 6A–D), a marked increase in the radiopacity with osteotomes can be seen and it indicates extensive bone deposition/compression. Instead, the osteotomy prepared with piezoelectric tips shows an underprepared site. Drills present an irregular osteotomy pattern consistent with bone removal/cutting and not a deposition. Osseodensification shows a constant narrow zone of bone deposition throughout the prepared osteotomy, without signs of microfractures and bone necrosis.

#### Bone to implant contact (BIC)

The highest BIC% was seen in osteotome (39.83 ± 3.44) followed by piezo (34.81 ± 3.78), osseodensification (33.26 ± 3.57), and conventional drills (30.73 ± 1.81). Statistically significant differences were not found only in the comparisons between osseodensification and drills, and between osseodensification and piezo, while all the other differences in BIC % resulted significant (Table 5).

**Table 5:** (A) Mean values and standard deviations of the bone to implant contact for the four techniques; (B) Post hoc comparison among the four different techniques, regarding the bone to implant contact

A Groups	Mean ± standard deviation
Osteotomes	39.83 ± 3.44
Osseodensification	33.26 ± 3.57
Piezo	34.81 ± 3.78
Drills	30.73 ± 1.81

B Compared group	Reference group	Mean difference
Osteotomes	Osseodensification	6.57*
	Piezo	5.02*
	Drill	9.10*
Osseodensification	Osteotome	− 6.57*
	Piezo	− 1.55
	Drill	2.53
Piezo	Osteotome	− 5.02*
	Osseodensification	1.55
	Drill	4.08*
Drills	Osteotome	− 9.10*
	Osseodensification	− 2.53
	Piezo	− 4.08*

^*^Indicates statistically significant values (*p* < 0.05). Positive mean difference = Decrease in bone to implant contact from the compared group to the reference group. Negative mean difference = Increase in bone to implant contact from the compared group to the reference group

## Discussion

In the last years, implant treatment has become a reliable and standard of care as an aesthetic and functional treatment for tooth loss [30], and a recently published systematic review showed that a 10 year implant survival rate might reach 96% [31]. However, many factors could affect implant survival [32, 33], leading to patient discomfort, further costs and clinical procedures [34]. Among different variables, implant site preparation is a key point in successful implant osseointegration [35]. This is due to changes in bone temperature which have the potential to promote bone necrosis, representing a significant risk factor for failure of osseointegration [36]. In this study, temperature changes were recorded by thermocouples and indicated minimum variations for conventional drilling and osteotomes at different depths. Piezoelectric system showed the highest change, with a statistically significant increase at T1, followed by a decrease at T2 and T3. The osseodensification system also showed a similar temperature change with an initial increase followed by a decrease in the temperature, but these changes—though greater than with the conventional drills and the osteotome—were significantly lower than the ones registered using the piezoelectric system.

Quaranta et al. [37] compared compressive osteotome vs conventional drills in porcine bone and reported lower temperatures in the osteotome group. In a study comparing the conventional drills and piezoelectric system in porcine bones [38], it was found that there was no difference between the two techniques in terms of heat generation. On the other hand, our findings are in consonance with those of Matys et al. [39] and Lajolo et al. [40] who reported significantly higher temperatures with the piezoelectric system. Moreover, regarding the osseodensification system, similar results emerged in the study by Huwais and Meyer [25] who reported a 3 °C rise with conventional drills as compared to 6 °C with osseodensification.

A number of studies have defined 47 ℃ as a critical temperature for the success of dental implants [3, 36]. Therefore, the temperature of 47 °C is considered the threshold level for bone survival during the implant site preparation, and the drilling time should be kept below 1 minute [41]. While there were significant differences in the temperature noted at different heights, they cannot be considered relevant to the clinical practice as the temperatures never reached the critical temperature range as discussed above, although piezoelectric system showed the extreme variation in temperature.

In this study, it was also evaluated the effect of these instruments on the bone to implant contact and the architecture of bone surrounding the implant site. To our best knowledge, this is the first study comparing conventional drills, osteotomes, piezoelectric system and osseodensification technique altogether.

In Buchter et al. [42] there was no difference in primary stability of implants placed in sites prepared with conventional drills and osteotomes in a minipig model study. In another article, where dogs have been used as a model to compare conventional drills and osteotomes, Kim et al. [43] reported higher primary stability in the osteotome group at week 0 and week 3, however, there were no significant differences at week eight. No differences were also found when comparing conventional drills and piezoelectric systems [44]. Moreover, most clinical studies have reported no significant difference in ISQ scores when comparing conventional drilling and osteotome [45–47]. In our study, the average ISQ scores were above 70, thus indicating good primary stability in terms of RFA, with no statistical differences in all four groups which is in agreement with most of the animal and clinical studies.

In terms of insertion torque, our study showed statistically significant results with the highest value in the osseodensification system, followed by the piezoelectric system, followed by drills and osteotomes. These results were in accordance with previous studies [48, 49].

Trabeculae numbers were also evaluated. To our best knowledge, this is the first study comparing the trabeculae number using all four methods for implant site preparation. We found that the average trabecular number was the highest in the osteotome group followed by piezo and osseodensification while the conventional drills had the least. This can be attributed to drills removing bone while the other techniques compacted the bone along the walls of implant sites. Another important finding was that osteotomes exhibited extreme bone compression while osseodensification drills used in osseodensification mode showed consistent radio-opacity throughout the length of osteotomy showing bone deposition along the walls. Drills showed a cut pattern of trabeculae throughout the working length.

With respect to the total implant site bone volume, Trisi et al. reported an increase of 30% when osseodensification technique was used [48]. In our study, all the groups showed statistically significant increase in bone volume and there were no significant intergroup differences. Though not significant, among the four groups studied, osseodensification system showed the highest increase in 3D bone volume.

At last, bone to implant contact was evaluated. Conventional drills reported minimum bone to implant contact compared to osteotomes, while piezo and osseodensification systems showed higher bone to implant contact percentage. Current evidence still reports controversial results. Nkenke et al. [50] and Kim et al. [43] outlined that osteotomes led to the higher bone to implant contact percentage compared to conventional drilling; while several studies comparing piezoelectric with conventional drills, found no difference in the bone to implant contact [42, 44, 51–54].

On the other hand, in one study Trisi et al. [48] compared the osseodensification system with the conventional drills and reported that the osseodensification system had higher values for the bone to implant contact.

## Conclusion

Within the limitations of this study (ex vivo model), the results did not find a significant difference among all four preparation techniques in terms of primary stability, however, the osseodensification group and the piezoelectric system showed significantly higher insertion torque than the others. Therefore, when placing an immediately loaded implant, osseodensification and piezo might provide more chances of immediate or early loading of implants, where a minimum insertion torque is required. Moreover, regarding the bone microstructure changes, there was an increase in trabeculae number, thus leading to a high bone to implant contact percentage. Even though osteotomes showed a higher bone compression, osseodensification showed a consistent deposition along the walls of osteotomy without over compressing the bone which may impair the blood supply to the bone-implant interface. To summarize, all techniques were found safe in terms of bone overheating, and the piezoelectric system had more chances of increasing the bone temperature, especially in the first perforation that requires longer preparation time, therefore, it might require a more experienced clinician to be applied safely. Osseodensification showed promising results in terms of heat generation while supporting good bone deposition throughout the prepared osteotomy, evaluated by micro-CT.
